# MicroRNA-185-5p inhibits hepatic gluconeogenesis and reduces fasting blood glucose levels by suppressing G6Pase

**DOI:** 10.7150/thno.46882

**Published:** 2021-06-26

**Authors:** Hui Zheng, Jian Wan, Yi Shan, Xi Song, Jie Jin, Qing Su, Song Chen, Xinyuan Lu, Jialin Yang, Quanmin Li, Yuping Song, Bo Li

**Affiliations:** 1 Medical College of Soochow University, Suzhou, Jiangsu; PLA Rocket Force Characteristic Medical Center, Beijing, China.; 2 Department of Endocrinology and Metabolic Diseases, Minhang Hospital, Fudan University, Shanghai 201100, China.; 3 Department of Emergency and Critical Care Medicine, Shanghai Pudong New Area People's Hospital, Shanghai University of Medicine and Health Sciences, Shanghai 201299, China.; 4 Department of Emergency and ICU, Changzheng Hospital, Naval Medical University, Shanghai 200003, China.; 5 Department of Endocrinology and Metabolic Diseases, Xinhua Hospital, Shanghai Jiao Tong University School of Medicine, Shanghai 200092, China.

**Keywords:** Type 2 diabetes, hyperglycemia, hepatic gluconeogenesis, miR-185-5p, metformin

## Abstract

**Aims/hypothesis:** MicroRNAs (miRNAs) are known to contribute to many metabolic diseases, including type 2 diabetes. This study aimed to investigate the roles and molecular mechanisms of miR-185-5p in the regulation of hepatic gluconeogenesis.

**Methods:** MicroRNA high-throughput sequencing was performed to identify differentially expressed miRNAs. High-fat diet-induced obese C57BL/6 mice and *db/db* mice, a genetic mouse model for diabetes, were used for examining the regulation of hepatic gluconeogenesis. Quantitative reverse transcriptase PCR and Western blotting were performed to measure the expression levels of various genes and proteins. Luciferase reporter assays were used to determine the regulatory roles of miR-185-5p on G6Pase expression.

**Results:** Hepatic miR-185-5p expression was significantly decreased during fasting or insulin resistance. Locked nucleic acid (LNA)-mediated suppression of miR-185-5p increased blood glucose and hepatic gluconeogenesis in healthy mice. In contrast, overexpression of miR-185-5p in *db/db* mice alleviated blood hyperglycemia and decreased gluconeogenesis. At the molecular level, miR-185-5p directly inhibited G6Pase expression by targeting its 3'-untranslated regions. Furthermore, metformin, an anti-diabetic drug, could upregulate miR-185-5p expression to suppress G6Pase, leading to hepatic gluconeogenesis inhibition.

**Conclusions/interpretation:** Our findings provided a novel insight into the role of miR-185-5p that suppressed hepatic gluconeogenesis and alleviated hyperglycemia by targeting G6Pase. We further identified that the /G6Pase axis mediated the inhibitory effect of metformin on hepatic gluconeogenesis. Thus, miR-185-5p might be a therapeutic target for hepatic glucose overproduction and fasting hyperglycemia.

## Introduction

Hepatic gluconeogenesis, stimulated by pancreatic glucagon and adrenal glucocorticoids, is essential for maintaining blood glucose levels in a normal range during prolonged fasting 1. The rate of hepatic glucose production is tightly controlled by two key enzymes: phosphoenolpyruvate carboxykinase (PEPCK) and glucose-6-phosphatase (G6Pase) 2. However, elevated expression and/or activity of these enzymes has been observed in patients with type 2 diabetes 3, 4, indicating that enhanced gluconeogenesis plays a critical role contributing to hyperglycemia and glucose intolerance. It has been well-established that mRNA expressions of PEPCK and G6Pase are tightly regulated by several transcriptional factors and coregulators, such as FoxO1 5, HNF4α 6, CREB 7, GR 8, PGC-1α 9, CRTC2 10, SIRT1 11, and SRC-1 12. Recent studies using knockout mice have demonstrated that these genes are essential for the induction of gluconeogenic enzymes during fasting. However, these results also strongly suggested that additional factors are likely to be involved in regulating hepatic gluconeogenesis and glucose production. Especially, whether hepatic gluconeogenic genes could be regulated by post-transcriptional factors remains poorly understood.

MicroRNAs (miRNAs), a class of small non-coding RNAs, bind to target mRNAs, destabilizing them and suppressing their translation 13. In the case of the target mRNA destabilization, one strand of mature miRNA duplexes is incorporated into Argonaute (Ago) proteins to form miRNA-induced silencing complexes (miRISCs), which repress the expression of partially or entirely complementary target mRNAs 14, 15. miRNAs have been much appreciated as critical regulators in many human diseases, including neurological disorders, cardiovascular diseases, and tumorigenesis 16, 17. Importantly, dysregulation of several miRNAs, such as miR-802, miR-200, and miR-26, are reported to be strongly associated with the development of metabolic disorders 18-20. Several studies have indicated that the aberrant expression of hepatic miRNAs contributes to hepatic insulin resistance, gluconeogenesis, and glucose homeostasis maintenance 21-25. For instance, our previous studies demonstrated that miR199a-5p and miR-592 played an important role in hepatic insulin resistance and steatosis 26-28. Besides, miR-27a promotes insulin resistance and mediates glucose metabolism by targeting PPAR-γ-mediated PI3K/AKT signaling 29. was found to be downregulated in the plasma of diabetic patients and isolated pancreatic islets of diabetic mice. Overexpression of miR-185-5p enhanced insulin secretion of pancreatic β-cells, promoted cell proliferation, and protected pancreatic β-cells from apoptosis by targeting SOCS3 and regulating the Stat3 pathway 30. However, the potential involvement of miRNAs in the direct regulation of hepatic gluconeogenesis has not been fully investigated.

In the present study, we used microRNA sequencing and showed that miR-185-5p was significantly decreased in the livers of fasted mice and diabetic mice. Our functional studies further indicated that miR-185-5p could inhibit gluconeogenesis by directly targeting G6Pase. Also, we demonstrated that metformin, an anti-diabetic drug, suppressed hepatic gluconeogenesis by modulating the /G6Pase axis. Therefore, our findings might be beneficial for exploring new therapeutic targets for treating hyperglycemia and diabetes.

## Methods

### Human studies

Twenty male patients with type 2 diabetes and 20 healthy age-matched male controls were recruited from the Department of Endocrinology and Metabolism, Minhang Hospital, Fudan University (Shanghai, China). Subjects with the following conditions were excluded: cancer, abnormal liver function, abnormal renal function, and infectious diseases. The study protocol was approved by the Human Research Ethical Committee of Minhang Hospital. All subjects provided written informed consent. The general characteristics of type 2 diabetes and healthy patients are shown in Table S1.

### Mouse experiments

Male C57BL/6 mice aged 10 weeks and *db/db* mice aged 8 weeks were purchased from the Animal Research Center of Nanjing University. All mice were housed in a temperature- and light-controlled environment with a 12-h light and 12-h dark cycle. To generate HFD-induced obese mice, C57BL/6 mice were fed ad libitum with normal chow or HFD (D12492; Research Diets) for 12 weeks. The diabetic parameters of 12-week HFD fed mice and *db/db* mice are shown in Figure S1. For the fasting experiment, male C57BL/6 mice aged 10 weeks were fasted for 16 h. For refeeding, the mice were fasted for 16 h and refed for 4 h. For metformin treatment, *db/db* mice received daily a dose of 200 mg · kg^-1^ · day^-1^ (metformin group) or an equal volume of vehicle (Saline) by i.p injection for 14 days. The insulin tolerance test (ITT), glucose tolerance test (GTT), and pyruvate tolerance test (PTT) were carried out using the guidelines provided by the Mouse Metabolic Phenotyping Centers, Yale School of Medicine (MMPC; https://www.mmpc.org/). GTT was performed by intraperitoneal injection of D-glucose (Sigma-Aldrich) at a dose of 2.0 mg/g body weight after a 16-h fast. For ITT, mice were injected with regular human insulin (Eli Lilly & Company, Indianapolis, IN) at a dose of 0.75 units/kg body weight after a 6-h fast. For PTT, mice were injected with sodium pyruvate (Sigma-Aldrich) at a dose of 1.5 mg/g body weight after a 16-h fast. Blood glucose was measured using a portable blood glucose meter (LifeScan; Johnson & Johnson, New Brunswick, NJ). The animal protocol was reviewed and approved by the Shanghai Pudong New Area People's Hospital and Minhang Hospital committees.

### Hepatic TG measurements

For lipid determinations, homogenates from liver tissues were extracted with NP40. After evaporation of the organic solvent, each sample's triglyceride content was measured with the triglyceride measurement reagent (BioVision, Milpitas, CA, USA), according to the manufacturer's instructions.

### Alanine aminotransferase (ALT) and aspartate aminotransferase (AST) analysis

Blood was directly collected from the heart by a 2 mL syringe insertion. Serum was obtained from the centrifuged blood samples. ALT and (AST) levels were measured by an autoanalyzer (Sunrise, Austria).

### Cell culture and glucose production assays

HEK293T cells were purchased from the Cell Bank of Type Culture Collection, Chinese Academy of Sciences (CAS, Shanghai, China). Mouse primary hepatocytes (MPHs) were isolated from the livers of C57BL/6 mice aged 10 weeks by collagenase perfusion and then purified by centrifugation 27. Fresh hepatocytes were seeded in 6-well plates at a density of 5 x 10^5^ cells per well in attachment media (Science Cell, USA). The media were then replaced with DMEM (Gibco, USA) within 24 h. For MPHs treatments, 10 μM forskolin, or 100 nm DEX, or 10nM glucagon, or 10nM insulin for 6 h were used. For Actinomycin D assay, cells were treated with 10 μg/ml Actinomycin D for 0, 2, 4, 6, 8h. For Metformin treatment, we used 2mM Metformin to treat MPHs for 24h. For glucose production assay, cells were transfected with miR-185-5p mimics, antisense or negative controls for 24 h. Subsequently, the medium was replaced with DMEM supplemented with 2 mM sodium pyruvate and 20 mM sodium lactate. 4 h later, the medium was collected, and the glucose concentration was measured with a colorimetric glucose assay kit (GAGO20; Sigma-Aldrich). The readings were normalized to the total protein content determined from the whole-cell extracts.

### Plasmids and luciferase assays

The entire 3'-untranslated region of mouse G6Pase gene containing either the wildtype or mutated binding sites was cloned and inserted into a pRL-null vector (Promega, USA). For luciferase assays, HEK293T cells were co-transfected in 12-well plates in duplicate wells with G6Pase reporter vectors together with 100nM mimics or negative controls using Lipofectamine 3000 for 24 h (Invitrogen, USA). Luciferase activities were measured consecutively by using the Dual-Luciferase Reporter Assay System (Promega, USA).

### LNA and recombinant adenoviruses

Locked nucleic acids (LNA) targeting miR-185-5p were designed and synthesized as unconjugated and fully phosphorothiolated oligonucleotides by Qiagen (Shanghai, China). LNA were intravenously delivered to C57BL/6 male mice at a concentration of 20 mg/kg. Mice were injected on two consecutive days and euthanized 16 days after LNA administration. Recombinant adenoviruses containing miR-185-5p were generated using the pAd-Easy system according to the manufacturer's instructions. Viruses were diluted in PBS and administered via the tail vein injection using 1*10^9^ plaque-forming units per mouse. The sequences of miR-185-5p mimics and control, anti-miR-185-5p and anti-NC, miR-185-5p LNA and control LNA were as follow: mimic-miR-185-5p: 5'-UGGAGAGAAAGGCAGUUCCUGA-3'; anti-miR-185-5p: 5'-UCAGGAACUGCCUUUCUCUCCA-3'; anti-NC: 5'-UUUGUACUACACAAAAGUACUG-3'; miR-185-5p LNA: 5'-UCAGGAACUGCCUUUCUCUCCA-3'; control LNA: 5'-UUCUCCGAACGUGUCACGUTT-3'.

### Mouse microRNA sequencing

microRNA high throughput sequencing and subsequent bioinformatics analysis were done by Cloud-Seq Biotech (Shanghai, China). Briefly, the total RNA of each sample was used to prepare the miRNA sequencing library, which included the following steps: 1) 3'-adaptor ligation; 2) 5'-adaptor ligation; 3) cDNA synthesis; 4) PCR amplification; 5) size selection of ~150 bp PCR amplicons (corresponding to ~22nt miRNAs). The libraries were denatured as single-stranded DNA molecules, captured on Illumina flow cells, amplified *in situ* as clusters, and finally sequenced for 50 cycles on Illumina HiSeq sequencer following the manufacturer's instructions.

### Serum miRNAs determination

Whole blood should be separated into serum and cellular fractions within 2h. The blood samples were centrifuged at 1,200 for 10 min at 4°C, and the upper plasma was transferred to a 5mL tube, and then centrifuged at 12,000 g for 10 min at 4°C to remove cellular components. Then the sera were stored at -80 °C. For serum RNA isolation, equal volume of Trizol was used, and three steps of phenol/chloroform purification were added to remove proteins from serum. The A260/A280 should be 1.8-2.0. Since U6 and 5S rRNA were degraded in serum samples, the expression levels of target miRNAs were directly normalized to total RNA. For miRNA detection, total RNA was reverse-transcribed using miScript II RT Kit (QIGEN, Shanghai). Subsequently, qRT-PCR was measured using miScript SYBR Green PCR Kit (QIGEN, Shanghai) on Light Cycler 480 (Roche, Basel, Switzerland).

### RNA isolation and quantitative real-time PCR (qRT-PCR)

Total RNAs were isolated from tissues or cells using TRIzol (Invitrogen) according to the manufacturer's instructions. The purified RNA was reverse-transcribed by PrimeScript RT Master Mix (TAKARA, Japan). For quantification of the transcripts of genes of interest, qRT-PCR was performed using SYBR Green Premix Ex Taq (Takara Bio, Otsu, Japan) on Light Cycler 480 (Roche, Basel, Switzerland). For the mRNA and miRNAs from tissues and cells, GAPDH and U6 were used for the relative quantification, respectively. For the miRNAs from the serum, we used the total RNA for the relative quantification. The primers were list as Table S2.

### Western blots

Homogenized tissues and cells were lysed in RIPA buffer containing 1 × PBS, 1% NP40, 5mM EDTA, 0.1% sodium dodecyl sulfate (SDS), 1mM Na_3_VO_4_, 1% phenylmethanesulfonylfluoride, complete protease inhibitor cocktail (Sigma-Aldrich), and complete phosphatase inhibitors. The lysates were centrifuged at 12,000 g for 10 min at 4°C to remove the insoluble materials, and the supernatants were boiled in an SDS loading buffer. The boiled samples were separated by 10% SDS-polyacrylamide gel and electroblotted onto nitrocellulose transfer membranes (Whatman, GE Healthcare). The membranes were blocked with 5% milk and incubated with different antibodies, followed by incubation with secondary antibodies. The primary antibodies used in Western blotting included anti-PEPCK (sc-271029, Santa Cruz Biotechnology), anti-G6Pase (ab243319, Abcam), anti-PGC-1α (ab106814, Abcam), and anti-β-actin (ab6276, Abcam).

### Statistical analysis

All values are presented as mean ± SEM. Differences between two groups were analyzed by 2-tailed Student's *t*-test (GraphPad Prism 6.01). Comparisons between more than two groups were analyzed by 1-way ANOVA (GraphPad Prism 6.01) followed by the Student-Newman-Keuls test. Statistical significance is displayed as * *P* < 0.05, ** *P* < 0.01, or *** *P* < 0.001.

## Results

### Reduction of hepatic miR-185-5p expression during fasting

**Figure 1:**
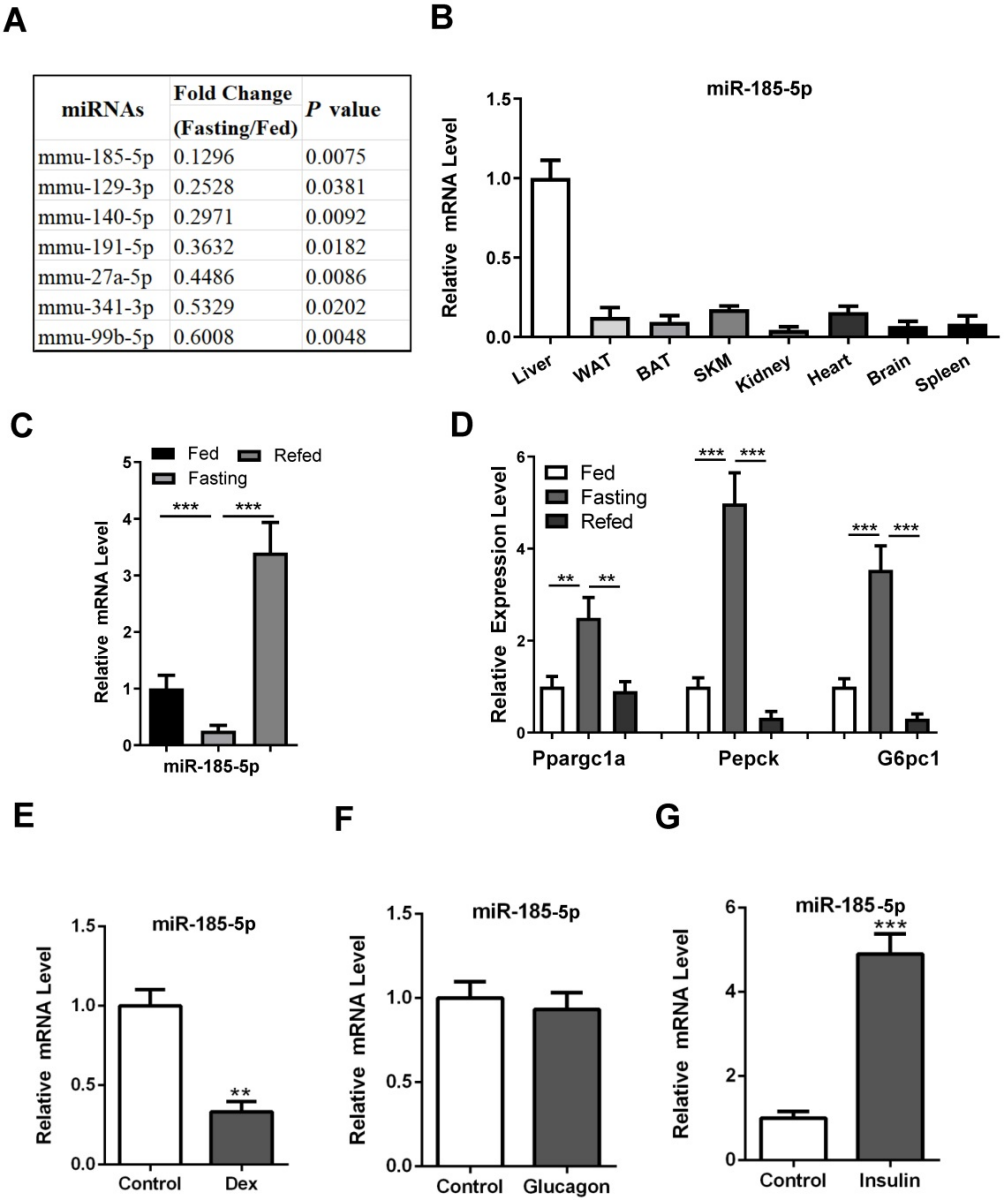
** Downregulation of hepatic miR-185-5p during fasting.** A: Expression of down-regulated miRNAs in the livers of C57BL/6 mice under fed or fasted states. B: Relative miR-185-5p expression in various mice organs, including liver, white adipose tissue (WAT), brown adipose tissue (BAT), skeletal muscle, kidney, heart, brain and spleen. C-D: qRT-PCR analysis of hepatic miR-185-5p and gluconeogenic gene expression in mice underfed, fasted, or refed states (n = 6). E-G: Relative expression of miR-185-5p in MPHs treated with DEX (100nM, E), glucagon (10nM, F), or insulin (10nM, G) for 6 h. n=4 per group. **P < 0.01, ***P < 0.001.

To explore the potential involvement of miRNAs in the regulation of hepatic gluconeogenesis, male C57BL/6 mice aged 10 weeks were subjected to fed or fasting conditions for 16 h. Subsequently, the miRNA expression in mouse liver was analyzed using miRNA high throughput sequencing. Expression levels of 28 miRNAs were significantly altered (Fold Change > 1.5, *P*-Value < 0.05), of which 21 were increased and 7 were decreased (Table S3). Since expression levels of many gluconeogenic genes, including PEPCK, G6Pase, Ppargc1a, and FoxO1, are known to be upregulated during fasting, we speculated that the corresponding miRNAs might be downregulated. Among them (Figure 1A), miR-185-5p was chosen because it was reported to regulate pancreatic β cell function and hepatic cholesterol homeostasis 31-33. Besides, the recent bioinformatic analysis showed that miR-185-5p was downregulated in Zucker diabetic fatty rats 34. These studies strongly suggested a potentially important role for miR-185-5p in controlling systemic glucose and/or lipid homeostasis.

Our results showed that miR-185-5p was enriched in the liver, while its expression in other tissues, including white adipose tissue and skeletal muscle, was relatively low (Figure 1B). As expected, miR-185-5p was substantially downregulated during fasting and induced upon refeeding (Figure 1C), consistent with a characteristic regulatory pattern for gluconeogenesis (Figure 1D).

### Regulation of miR-185-5p by glucocorticoid and insulin in hepatocytes

To explore a mechanism for the downregulation of miR-185-5p, mouse primary hepatocytes (MPHs) and Hep1-6 cells were treated with three stimuli known to mimic fasting or refed signals. As a result, dexamethasone, the analog of glucocorticoid, but not the glucagon, suppressed miR-185-5p expression significantly (Figure 1E-F and Figure S2A-B). Conversely, miR-185-5p expression was induced by insulin treatment (Figure 1G and Figure S2C). Time course experiments further showed that miR-185-5p inhibition by dexamethasone occurred at 6 h and peaked at 12 h in MPHs (Figure S2D). However, downregulation of miR-155, which has been shown to be directly suppressed by glucocorticoid 35, occurred at 2 h (Figure S2E). These data suggest that the negative regulation of miR-185-5p by dexamethasone likely requires synthesis of new proteins. In agreement with this hypothesis, pre-treatment with cycloheximide, a protein synthesis inhibitor, reversed the inhibitory effects of dexamethasone on miR-185-5p expression (Figure S2F).

### Suppression of miR-185-5p by FoxO1

**Figure 2:**
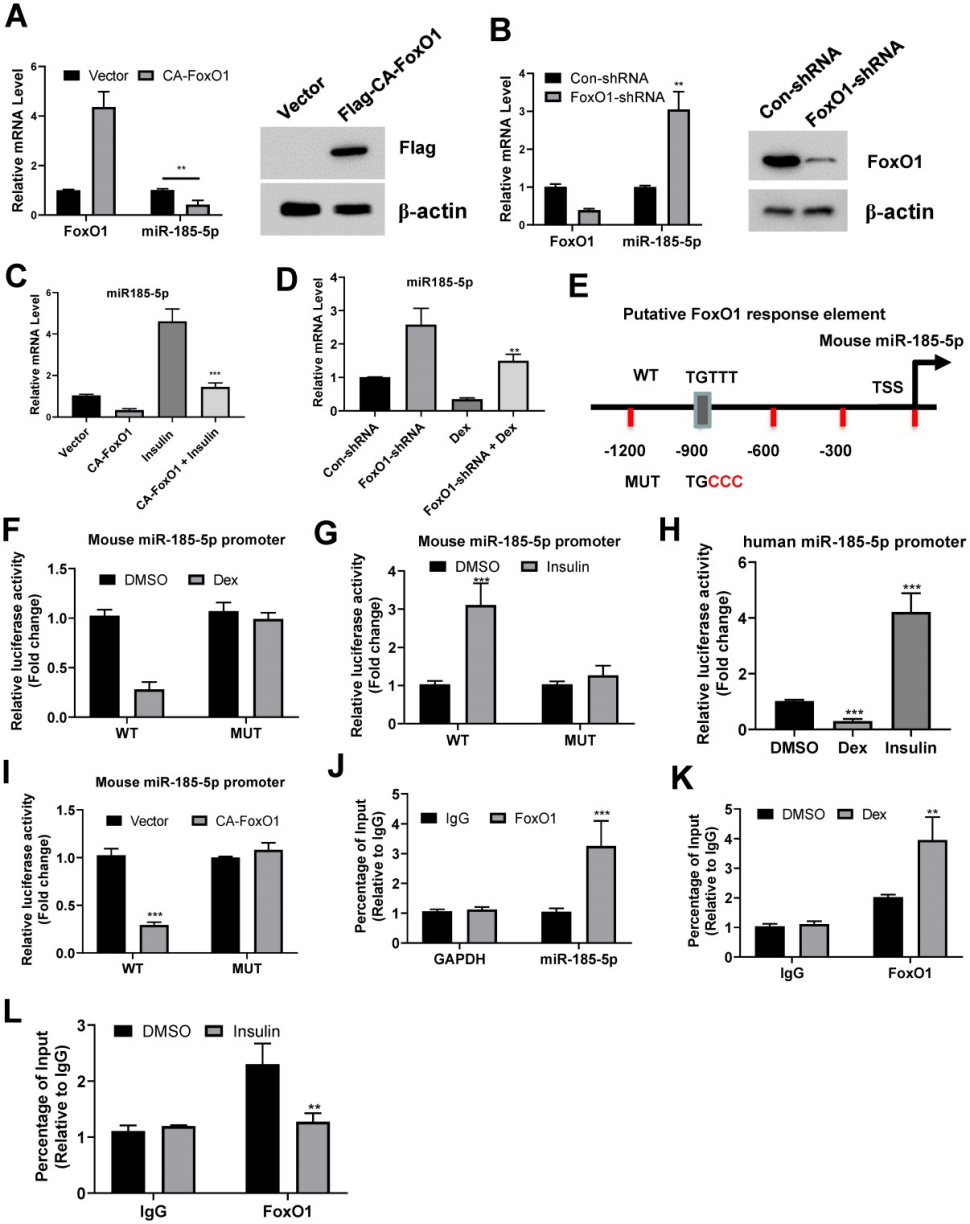
** Suppression of miR-185-5p by FoxO1.** A: MPHs were infected with adenovirus expressing a constitutively active FoxO1 (Flag-CA-FoxO1) or vector control. The mRNA expression levels of FoxO1 and miR-185 were measured by real-time PCR assay. The protein expression of Flag-CA-FoxO1 was examined by western blot assay. B: MPHs were transfected with con-shRNA or shRNA against FoxO1. The mRNA expression levels of FoxO1 and miR-185 were measured by real-time PCR assay. C: MPHs infected with adenovirus expressing a constitutively active FoxO1 (Flag-CA-FoxO1) or vector control were treated with insulin (10nM, C) for 6 h. The mRNA expression levels of miR-185 were measured by real-time PCR assay. The protein expression of endogenous FoxO1 was examined by western blot assay. D: MPHs transfected with con-shRNA or shRNA against FoxO1were treated with dexamethasone (100nM, Dex) for 6 h. The mRNA expression levels of miR-185 were measured by real-time PCR assay. E: Schematic diagram shows mouse miR-185 promoter and putative FoxO1 binding sites. TSS: transcription. The mutations were highlighted in red. F: Relative luciferase activity of the firefly reporter containing the wt or mutant mouse miR-185 promoter was detected in MPHs with or without dexamethasone treatment. G: Relative luciferase activity of the firefly reporter containing the wt or mutant mouse miR-185 promoter was detected in MPHs with or without insulin treatment. H: Relative luciferase activity of the firefly reporter containing the wt human miR-185 promoter was detected in HepG2 cells with or without dexamethasone or insulin treatment. I: Relative luciferase activity of the firefly reporter containing the wt or mutant mouse miR-185 promoter was detected in MPHs infected with adenovirus expressing a constitutively active FoxO1 (Flag-CA-FoxO1) or vector control. J: ChIP shows enrichment of FoxO1 at the mouse miR-185 promoter in MPHs. K: Dexamethasone treatment increased the occupation of FoxO1 at the mouse miR-185 promoter in MPHs. L: Insulin treatment decreased the occupation of FoxO1 at the mouse miR-185 promoter in MPHs. **P < 0.01, ***P < 0.001.

Recent studies demonstrated that the transcription factor FoxO1 is essential for glucocorticoid- and insulin-dependent regulation of certain genes during fasting and refed conditions. FoxO1 was shown to activate G6pase and suppress Glucokinase in the liver, respectively. We thus tested whether FoxO1 was also involved in the transcriptional repression of miR-185-5p. Forced expression of adenovirus expressing a constitutively active FoxO1 (CA-FoxO1) significantly reduced expression of miR-185-5p in MPHs and Hep1-6 cells (Figure 2A and Figure S3A). In contrast, knockdown of FoxO1 in both cells resulted in a dramatic induction of miR-185-5p (Figure 2B and Figure S3B). Additionally, overexpression of CA-FoxO1 blocked the induction of miR-185-5p by insulin (Figure 2C and Figure S3C), while dexamethasone-dependent suppression of miR-185-5p was largely attenuated by FoxO1 depletion (Figure 2D and Figure S3D). These data suggest the presence of a mechanism for glucocorticoid-mediated suppression of miR-185-5p during fasting which requires FoxO1.

We then analyzed the promoter region of mouse miR-185-5p and found a consensus sequence for FoxO1 binding site (Figure 2E). Therefore, to test whether FoxO1 could regulate miR-185-5p promoter activity, Hep1-6 cells were transfected with a luciferase reporter construct containing the promoter. As expected, the miR-185-5p promoter activity was inhibited by dexamethasone and increased by insulin (Figure 2F-G). Similar results were also observed in human miR-185-5p promoter (Figure 2H). However, point-mutation of FoxO1 binding site completely blocked the regulatory roles of dexamethasone and insulin (Figure 2E-F and Figure S3E-F). Moreover, the wild-type miR-185-5p promoter activity, but not the mutant, was markedly decreased in cells co-transfected with the CA-FoxO1 expression plasmid (Figure 2I). In addition, our chromatin immunoprecipitation assays showed that endogenous FoxO1 protein was associated with the predicted regions of mouse promoter (Figure 2J). This association could be enhanced and disrupted by dexamethasone and insulin treatment, respectively (Figure 2K-L). Taken together, our results indicate that FoxO1 could inhibit miR-185-5p expression. Besides, the abilities of glucocorticoid and insulin to decrease and increase miR-185-5p expression in hepatocytes are dependent on FoxO1.

### Down-regulation of miR-185-5p in diabetic mice and patients

**Figure 3:**
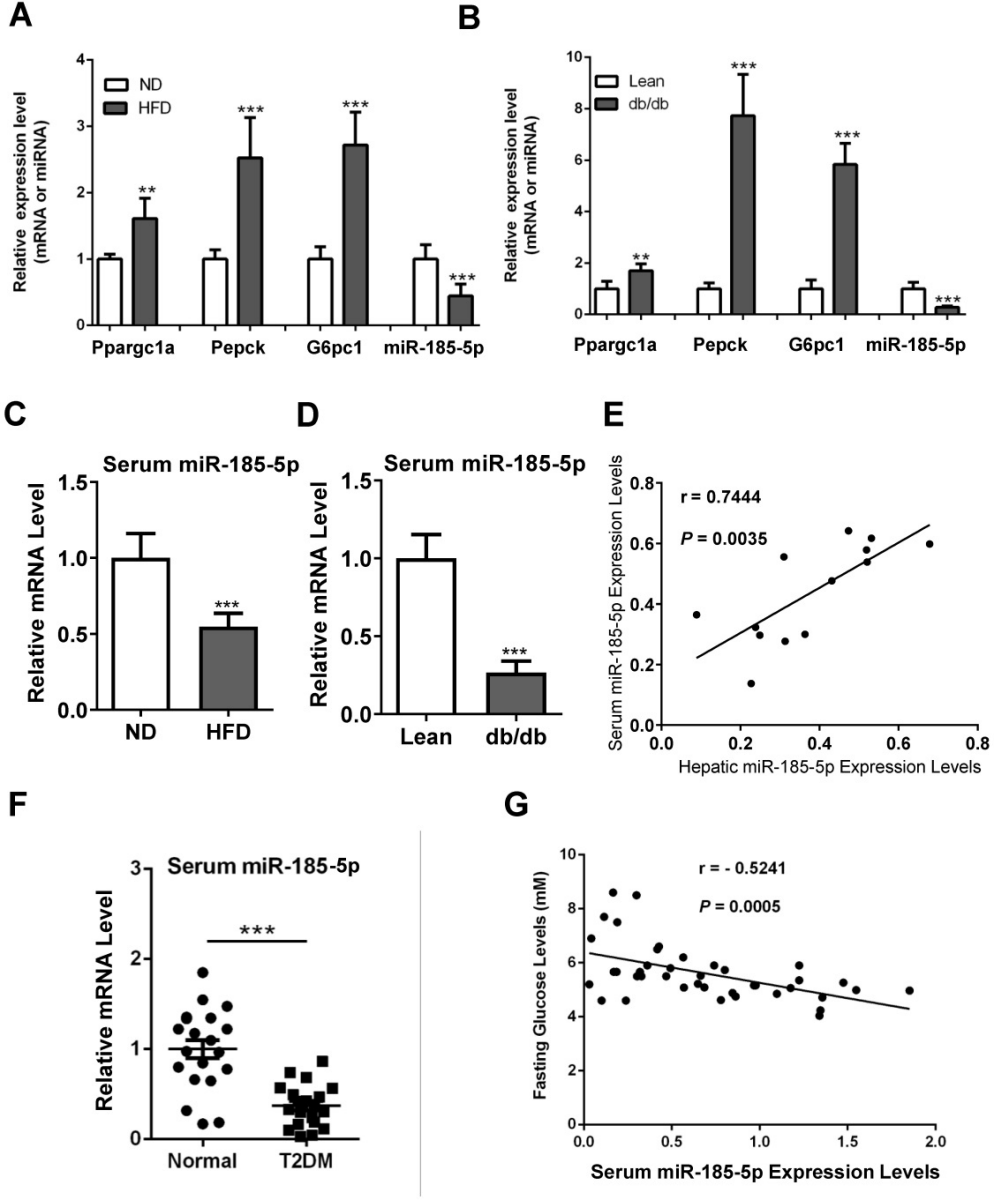
** Reduced expression of miR-185-5p in diabetes.** A: qRT-PCR analysis of hepatic miR-185-5p or gluconeogenic gene expression in mice fed a high-fat diet or normal diet for 12 weeks. n=6 per group. B: qRT-PCR analysis of hepatic miR-185-5p or gluconeogenic gene expression in lean or *db/db* mice. n=6 per group. C: Relative miRNA expression of serum miR-185-5p from mice in A. D: Relative miRNA expression of serum miR-185-5p from mice in B. E: Pearson R- and P-value for normalized serum miR-185-5p mRNA levels versus hepatic miR-185-5p mRNA levels in mice. n=13 per group. F: Relative miRNA expression of serum miR-185-5p from normal subjects and diabetic patients. n=20 per group. G: Pearson R- and P-value for normalized serum miR-185-5p mRNA levels versus fasting blood glucose levels in human subjects. n=40. **P < 0.01, ***P < 0.001.

Hepatic gluconeogenesis is usually increased in diabetic subjects, contributing to hyperglycemia. Therefore, we examined the miR-185-5p expression in the livers of diabetic mice. As expected, gluconeogenic genes, including Ppargc1a, PEPCK, and G6Pase, were increased in the livers of mice fed HFD for 12 weeks, compared to those fed a normal diet (Figure 3A). Similar results were also observed in the leptin receptor-deficient *db/db* mice, a genetic mouse model for diabetes (Figure 3B). It has been shown that miR-185-5p can be secreted into the blood, and changes of serum miRNAs serve as a noninvasive biomarker for many types of human diseases. We found that serum levels of miR-185-5p were reduced in HFD-induced obese mice and *db/db* mice (Figure 3C-D). Besides, there was a significant correlation between hepatic miR-185-5p expression and serum miR-185-5p concentrations in diabetic mice (Figure 3E).

We recruited a small cohort of normal subjects and patients with type 2 diabetes. The qRT-PCR analysis showed that serum miR-185-5p concentrations were lower in diabetic patients than normal controls (Figure 3F). Pearson correlation analysis revealed a significant and negative correlation between serum miR-185-5p concentrations and fasting blood glucose levels (Figure 3G). These results suggested that miR-185-5p is involved in the post-transcriptional regulation of hepatic gluconeogenesis.

### Inhibition of miR-185-5p by LNA increases blood glucose and hepatic gluconeogenesis

**Figure 4:**
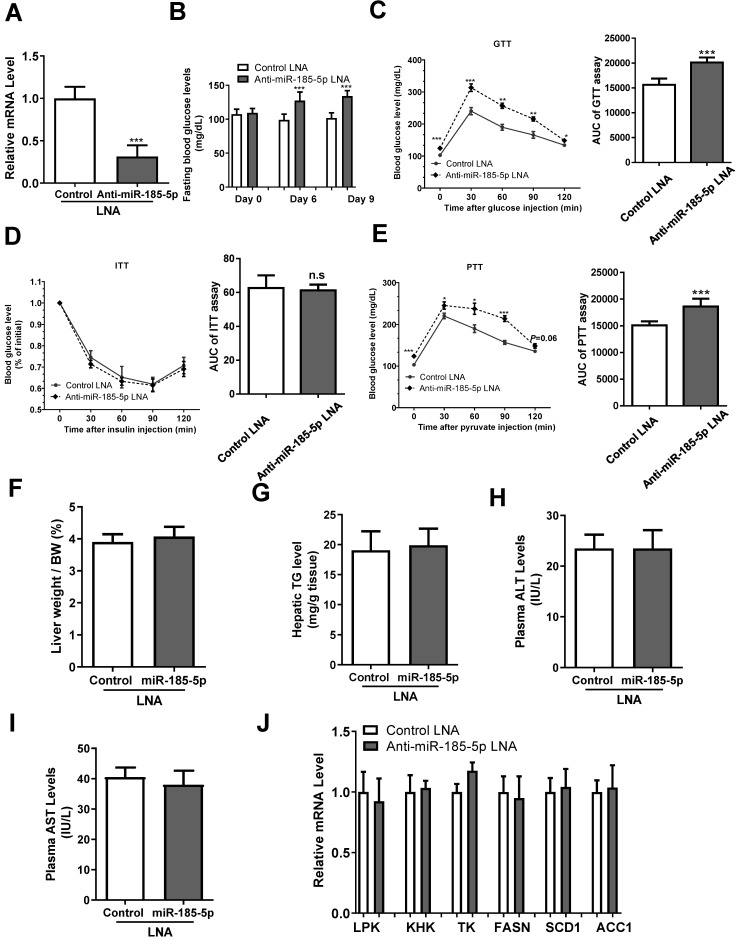
** miR-185-5p regulates hepatic gluconeogenesis *in vivo*.** Male C57BL/6J WT mice were injected with miR-185-5p LNA or the negative control via the tail vein. n=8 per group. A: Measurement of hepatic miR-185-5p expression. B: Examination of fasting blood glucose. C-E: Performance GTTs (C), ITTs (D), and PTTs (E), at day 10, 12, and 14, respectively. The AUC of glycemia was also calculated. F-I: Measurement of hepatic liver weight/body weight ratio (F), hepatic TG level (G), plasma ALT and AST levels (H-I). J: Measurement of mRNA levels of glycolytic and lipogenic enzymes. *P < 0.05, **P < 0.01, ***P < 0.001.

To investigate the effect of miR-185-5p on hepatic gluconeogenesis, we injected anti miR-185-5p- LNA or control LNA into male C57BL/6 mice. The anti-miR-185-5p LNA significantly inhibited the hepatic miR-185-5p expression level compared with control LNA injection (Figure 4A), and increased fasting blood glucose levels at days 6 and 9 after injection (Figure 4B). Mice with anti-miR-185-5p LNA injection also exhibited increased hepatic gluconeogenesis and glucose intolerance as determined by GTT and PTT, respectively (Figure 4C,4E), without changes in insulin sensitivity as determined by ITT (Figure 4D); the area under the curve (AUC) of glycemia was also calculated (Figure 4C-E). Furthermore, the liver weight/body weight ratio, hepatic TG level, plasma ALT, and AST levels were not affected by anti- LNA (Figure 4F-I). Also, expression levels of genes related to glycolysis and lipogenesis were not affected by anti- miR-185-5p LNA (Figure 4J). Together, these results demonstrated a critical and specific role of miR-185-5p in the regulation of hepatic gluconeogenesis.

### miR-185-5p regulates hepatic gluconeogenesis by targeting G6Pase

**Figure 5:**
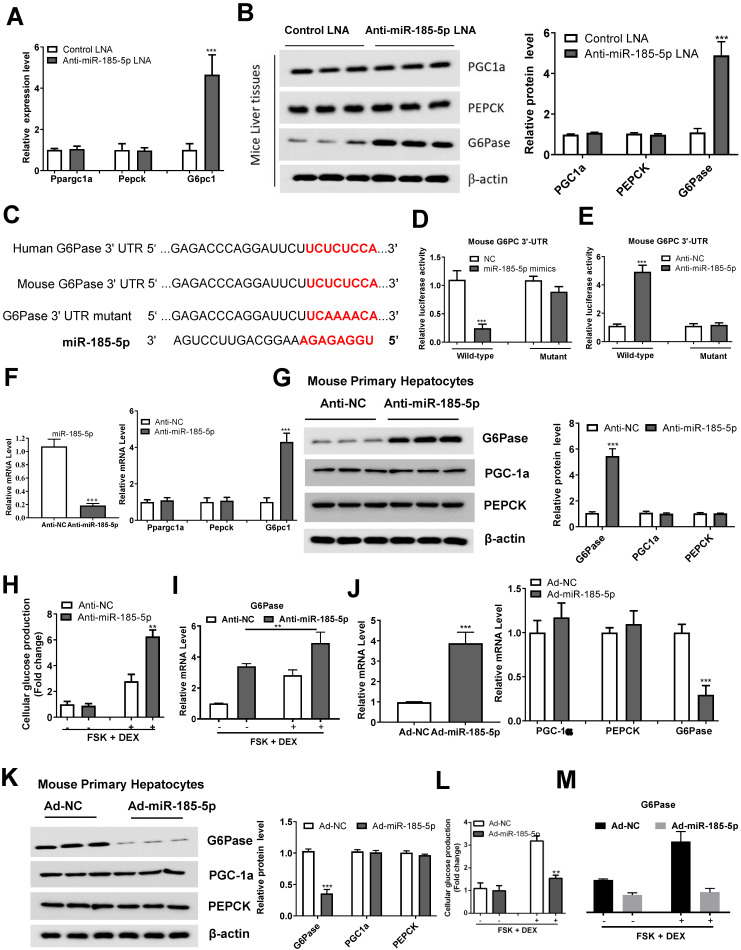
** miR-185-5p directly regulates G6Pase expression.** A-B: mRNA (A) and protein (B) expression of gluconeogenic genes (Ppargc1a, PEPCK, and G6Pase) in the livers of C57BL/6J mice administered with miR-185-5p LNA or negative control. The quantification plot was based on scanning densitometry analysis using the ImageJ software (v 1.8.0). C: Sequence alignment of miR-185-5p with the 3'-UTR of the mouse and human G6Pase. D-E: HEK293T cells were co-transfected with wildtype or mutant 3ʹ-UTR reporter plasmids of G6Pase with miR-185-5p mimics (D), miR-185-5p antisense (E). F-G: MPHs were transfected with miR-185-5p antisense or negative control for 48 h and then treated with FSK (10 μM) and DEX (100 nm) for an additional 6 h. Then, mRNA levels of miR-185-5p and gluconeogenic genes (Ppargc1a, PEPCK, and G6Pase) were examined (F) and G6Pase protein level was determined (G); the quantification plot was based on scanning densitometry analysis using the ImageJ software (v 1.8.0). H-I: MPHs were transfected with miR-185-5p antisense or negative control for 48 h and then treated with FSK (10 μM) and DEX (100 nm) for an additional 6 h. Then, cellular glucose production (H) and G6Pase mRNA levels (I) were determined. J-K: MPHs were infected with Ad-miR-185-5p or negative control for 48 h and then treated with FSK (10 μM) and DEX (100 nm) for additional 6 hours. Then, mRNA (J) and protein (K) levels of gluconeogenesis (PGC-1α, PEPCK, and G6Pase) were examined. L-M: MPHs were infected with Ad-miR-185-5p or negative control for 48 h and then treated with FSK (10 μM) and DEX (100 nm) for an additional 6 h. Then, cellular glucose production and G6Pase mRNA levels (L) and G6Pase mRNA levels (M) were determined. **P < 0.01. ***P < 0.001.

We identified the expression of effector genes downstream of miR-185-5p, involved in gluconeogenesis, by performing qRT-PCR and Western blotting. We found that miR-185-5p inhibition in mice significantly upregulated G6Pase, without affecting Ppargc1a and PEPCK expression (Figure 5A-B). Importantly, we identified a potential miR-185-5p binding site within 3ʹ- UTR of G6Pase, suggesting G6Pase might be a downstream target of miR-185-5p (Figure 5C). To test this possibility, we co-transfected the psiCHECK2-promoter-based G6Pase 3ʹ- UTR reporter with miR-185-5p mimics, anti-miR-185-5p, or the negative control in HEK293T cells. The luciferase reporter assay revealed that miR-185-5p mimics reduced, while anti- increased the luciferase activity in HEK293T cells (Figure 5D-E). In contrast, mutation of the miR-185-5p target sites abrogated miR-185-5p-dependent regulation in luciferase activity (Figure 5D-E), suggesting a direct interaction of miR-185-5p with G6Pase 3'-UTR.

To better understand the role of miR-185-5p in regulating hepatic gluconeogenesis, MPHs were transfected with anti-miR-185-5p or anti-miR NC, and the transfection efficiency was examined. As shown in Figure 5F, anti-miR-185-5p transfection selectively increased G6Pase mRNA level without altering the Ppargc1a and PEPCK expression. Similarly, G6Pase protein levels were also increased in response to anti-miR-185-5p transfection (Figure 5G). MPHs were also transfected with anti-miR-185-5p or control miR-NC and then treated with forskolin (FSK) and DEX to stimulate glucose production. Figure 5H shows that inhibition of miR-185-5p significantly promoted glucose production in MPHs in the presence of FSK/DEX, simultaneously increasing the G6Pase mRNA expression (Figure 5I). On the contrary, following Ad-miR-185-5p adenoviruses transfection (Figure 5J), MPHs exhibited decreased G6Pase mRNA and protein levels (Figure 5J-K). The glucose production was also reduced in the presence of FSK/DEX compared to control adenoviruses (Ad-NC) (Figure 5L) accompanied by decreased G6Pase mRNA (Figure 5M). To understand the reduction in G6Pase mRNA by miR-185-5p, MPHs infected with Ad-NC or Ad-miR-185-5p adenoviruses were treated with Actinomycin D to inhibit transcription. G6Pase mRNA readily disappeared from Ad-miR-185-5p-expressing cells, indicating increased G6Pase mRNA degradation (Figure S4A).

Importantly, silencing the G6Pase expression largely abolished the role of anti-miR-185-5p in the regulation of glucose production (Figure S4B-C). Glucocorticoid receptor (GR) is known to be the DEX target, and its depletion significantly prevented DEX-induced miR-185-5p repression and G6Pase expression (Figure S5A-B), suggesting that GR is upstream of the miR-185-5p-G6Pase axis. Collectively, these results demonstrated that miR-185-5p regulated hepatic gluconeogenesis *in vitro*.

### miR-185-5p overexpression improves hyperglycemia and inhibits gluconeogenesis in db/db mice

**Figure 6:**
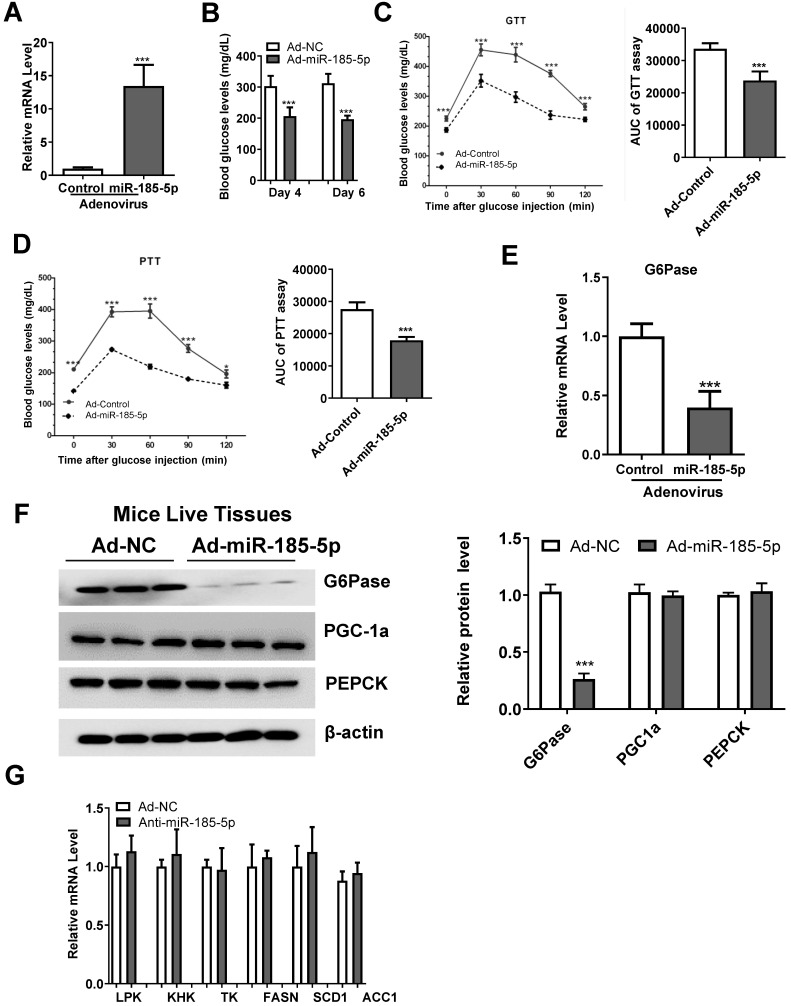
** Hepatic overexpression of miR-185-5p improves hyperglycemia in *db/db* mice.** Male *db/db* mice were infected with adenovirus-overexpressing miR-185-5p (Ad-miR-185-5p) or negative control (Ad-NC) via tail-vein injection. n=8 per group. A: Expression of hepatic miR-185-5p expression by qRT-PCR at day 15 post-injection. B: Fasting blood glucose levels at days 4 and 6. C-D: GTT and PTT at days 8 and 11. The AUC of glycemia was also calculate. E-F: mRNA and protein levels of G6Pase in the livers of mice. The quantification plot was based on scanning densitometry analysis using the ImageJ software (v 1.8.0). G: mRNA levels of glycolytic and lipogenic enzymes were determined by qRT-PCR. *P < 0.05, ***P < 0.001.

To explore the role of miR-185-5p in hepatic gluconeogenesis *in vivo*, Ad-miR185 adenoviruses or control scrambled adenoviruses (Ad-miR-NC) were injected into male *db/db* mice via the tail vein. As is evident from Figure 6A, the hepatic miR-185-5p level was significantly increased in mice infected with Ad-miR-185-5p viruses compared with Ad-miR NC viruses. Compared to the levels in the Ad-miR NC group, Ad-miR-185-5p mice exhibited lower fasting blood glucose levels at days 4 and 6 (Figure 6B), improved glucose tolerance (Figure 6C) and decreased gluconeogenesis in the liver (Figure 6D). Consistent with these results, the mRNA and protein expression levels of G6Pase were also reduced (Figure 6E-F). However, mRNA levels of glycolytic and lipogenic enzymes were not affected by miR-185-5p overexpression (Figure 6G). Thus, these results demonstrated that overexpression of miR-185-5p could improve blood glucose and suppress gluconeogenesis in *db/db* mice to alleviate diabetes.

### Metformin decreases G6Pase expression by upregulating miR-185-5p

**Figure 7:**
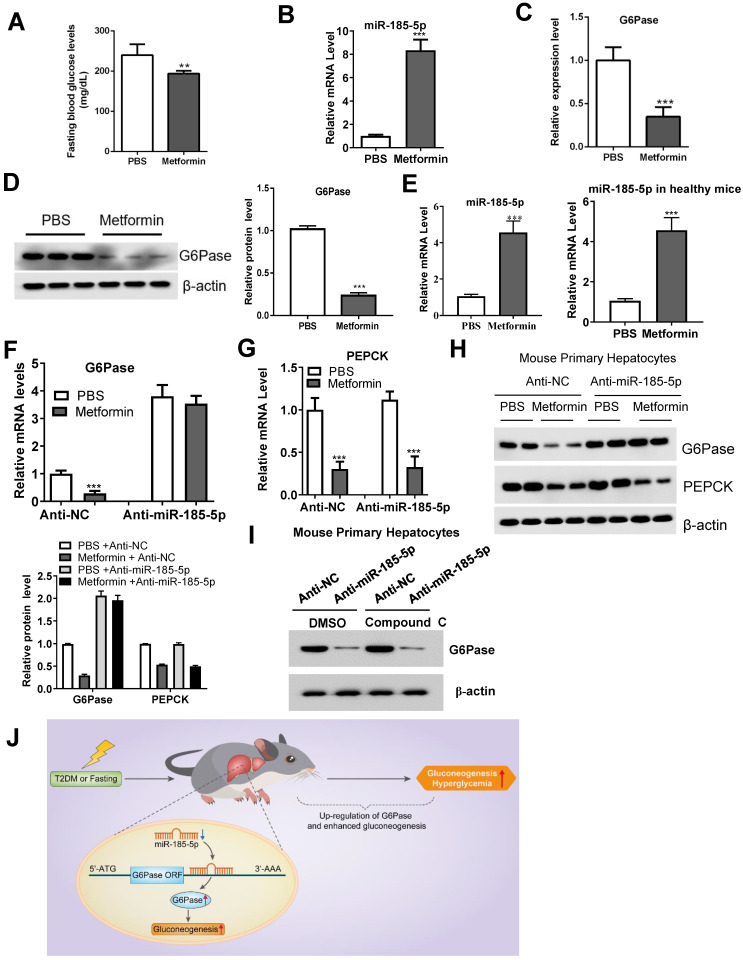
** Metformin inhibits G6Pase expression by targeting miR-185-5p.** *db/db* mice were daily treated with metformin (200 mg/kg) or vehicle control by i.p. injection for 14 days. A-D: Fasting blood glucose levels (A), hepatic miR-185-5p expression (B), mRNA and protein levels of G6Pase in the livers (C-D), were analyzed in two groups of mice. n=5 per group. E: Relative expression of miR-185-5p in MPHs (left panel) or health mice (right panel) treated with metformin or vehicle control for 24 h. F-H: MPHs were treated with miR-185-5p antisense or negative control for 24 h, and then treated with metformin or vehicle control for another 24 h. Then, mRNA (F-G) and protein (H) levels of PEPCK and G6Pase were analyzed. The quantification plot was based on scanning densitometry analysis using the ImageJ software (v 1.8.0). ** *P* < 0.01, *** *P* < 0.001. I: MPHs were transfected with miR-185 antisense or negative control for 36 h and then treated with DMSO or Compound C for additional 12 h. Then, the protein level of G6Pase was determined. J: Schematic model: We propose that miR-185-5p could suppress hepatic gluconeogenesis and alleviate hyperglycemia by targeting the miR-185-5p/G6Pase axis.

The biguanide compound metformin is used as first-line therapy for T2DM 36 and acts primarily by suppressing hepatic gluconeogenesis 37, 38. However, the molecular mechanism that underlies this effect remains a subject of active investigation. Metformin treatment decreased the fasting blood glucose in *db/db* mice (Figure 7A). Interestingly, compared to the control group, metformin treatment also upregulated hepatic miR-185-5p expression in *db/db* mice liver (Figure 7B), and reduced hepatic G6Pase mRNA and protein expression (Figure 7C-D). Metformin treatment also led to a significantly increased miR-185-5p expression in MPHs and healthy mice (Figure 7E). To get a better insight into the role of miR-185-5p in the regulation of G6Pase by metformin, anti-miR-185-5p was transfected into MPHs and then treated with or without metformin. We found that metformin-dependent inhibition of G6Pase mRNA expression was largely reversed by anti-miR-185-5p (Figure 7F). However, inhibition of miR-185-5p did not influence metformin-dependent PEPCK mRNA reduction (Figure 7G). Consistent with the mRNA level, anti-miR-185-5p rescued metformin-suppressed G6Pase protein expression (Figure 7H). In contrast, metformin-mediated PEPCK protein expression was not affected by anti-miR-185-5p (Figure 7H), supporting the notion that metformin specifically decreases the expression of G6Pase via miR-185-5p. However, Compound C, an antagonist of AMPK signaling, could not affect the role of anti-miR-185-5p, suggest that metformin-miR-185-5p regulatory axis might be independent of AMPK signaling (Figure 7I). To determine whether miR-185-5p is required for metformin's glucose-lowering effect, *db/db* mice were daily treated with metformin (200 mg/kg) or vehicle control by i.p. injection for 3 weeks then injected with control LNA or miR-185-5p LNA for additional 6 days. We found that metformin treatment significantly inhibited gluconeogenesis in diabetic mice injected with control LNA. However, metformin's glucose-lowering effect was largely attenuated in mice treated with miR-185-5p LNA (Figure S6A-C). These data indicated that anti-miR-185-5p could reverse the inhibitory effect of metformin on gluconeogenesis in diabetic mice.

## Discussion

Although it has been well-established that enhanced hepatic gluconeogenesis is associated with fasting hyperglycemia and type 2 diabetes 39, the molecular mechanisms remain poorly understood. Gluconeogenesis contributes approximately half of the total hepatic glucose production in humans following an overnight fast and is primarily responsible for increasing fasting hepatic glucose production in individuals with type 2 diabetes 40-44. Major gluconeogenic precursors, including lactate, alanine, and glycerol, are subjected to diverse regulatory mechanisms. Besides, hepatic gluconeogenesis is indirectly regulated by lipolysis, hormonal and neural control of hepatic glucose production, and gluconeogenic capacity of the liver 2. The best-characterized pathway for insulin-dependent transcriptional control of gluconeogenic gene expression involves members of the FOXO family of transcription factors (FOXO1, FOXO3a, and FOXO4) 45. Gain-of-function or loss-of-function perturbations of the FOXO-PGC1α axis have marked effects on G6Pase and PEPCK protein levels and glycemia in rodent studies 5, 9.

It has been reported that miR-185-5p could regulate insulin secretion and pancreatic β cell viability. Thus, miR-185-5p might play a role in type 1 diabetes, in which the ability of pancreatic β cells to produce insulin is compromised, and β-cell death is the final and critical step in the development of the disease. In the present study, we provided evidence to support the novel and critical role of miR-185-5p in hepatic gluconeogenesis. Our findings demonstrated that miR-185-5p was down-regulated during fasting. We also showed that miR-185-5p inhibited G6Pase expression by directly binding to its 3'-UTR and reduced hepatic glucose output. Restoring miR-185-5p expression in diabetic mice liver significantly suppressed hepatic gluconeogenesis and alleviated hyperglycemia. We further showed that metformin inhibited hepatic gluconeogenesis by targeting the miR-185-5p/G6Pase axis. Our study identified miR-185-5p as a novel regulator in hepatic gluconeogenesis and restoring its expression in diabetic mice might be a novel therapeutic strategy for treating diabetes. Our gene delivery approach was liver-targeted and showed that the inhibitory effect of miR-185-5p on glucose production and clearance in *db/db* mice might mainly be mediated by hepatic G6pase expression. Nevertheless, miR-185-5p-mediated insulin secretion and pancreatic β cell viability may also contribute to its function in whole-body glucose homeostasis.

Metformin could affect many cellular signaling pathways, including activation of LKB1-AMPK signaling, to regulate the expression of gluconeogenic genes 46, 47. Our study observed that metformin specifically reduces the expression of G6Pase through miR-185-5p without affecting PEPCK, indicating the specificity of miR-185-5p on the metformin-G6Pase regulatory axis. We also found that metformin-dependent G6Pase mRNA and protein expression inhibition was largely reversed by anti-miR-185-5p. However, Compound C, an antagonist of AMPK signaling, could not affect the role of anti-miR-185-5p. Therefore, our results suggest that metformin-miR-185-5p regulatory axis might be independent of AMPK signaling.

There are still outstanding issues that remain to be clarified. Although the adenovirus-mediated infection has been widely utilized in liver research 32, 36, future studies using liver-specific miR-185-5p knockout mice are still needed. Also, the sample size in our present study was relatively small. Further studies using larger populations are needed to confirm the relationship between serum miR-185-5p expression and metabolic parameters.

In summary, our findings provided a novel insight into miR-185-5p's role in suppressing hepatic gluconeogenesis and alleviating hyperglycemia. We further found that metformin inhibited hepatic gluconeogenesis by targeting the miR-185-5p/G6Pase axis. miR-185-5p might, therefore, be a therapeutic target for hepatic gluconeogenesis-induced diabetes (Figure 7J).

## Supplementary Material

Supplementary figures and tables.
