# Supplementary material for: MicroRNA-185-5p inhibits hepatic gluconeogenesis and reduces fasting blood glucose levels by suppressing G6Pase
Source: Theranostics. 2021 Jun 26;11(16):7829–43. doi: 10.7150/thno.46882 (PMC8315058; doi:10.7150/thno.46882)
Supplement: Supplementary file 1 — Supplementary figures and tables. [file thnov11p7829s1.pdf]

Supplementary Figures

Figure S1

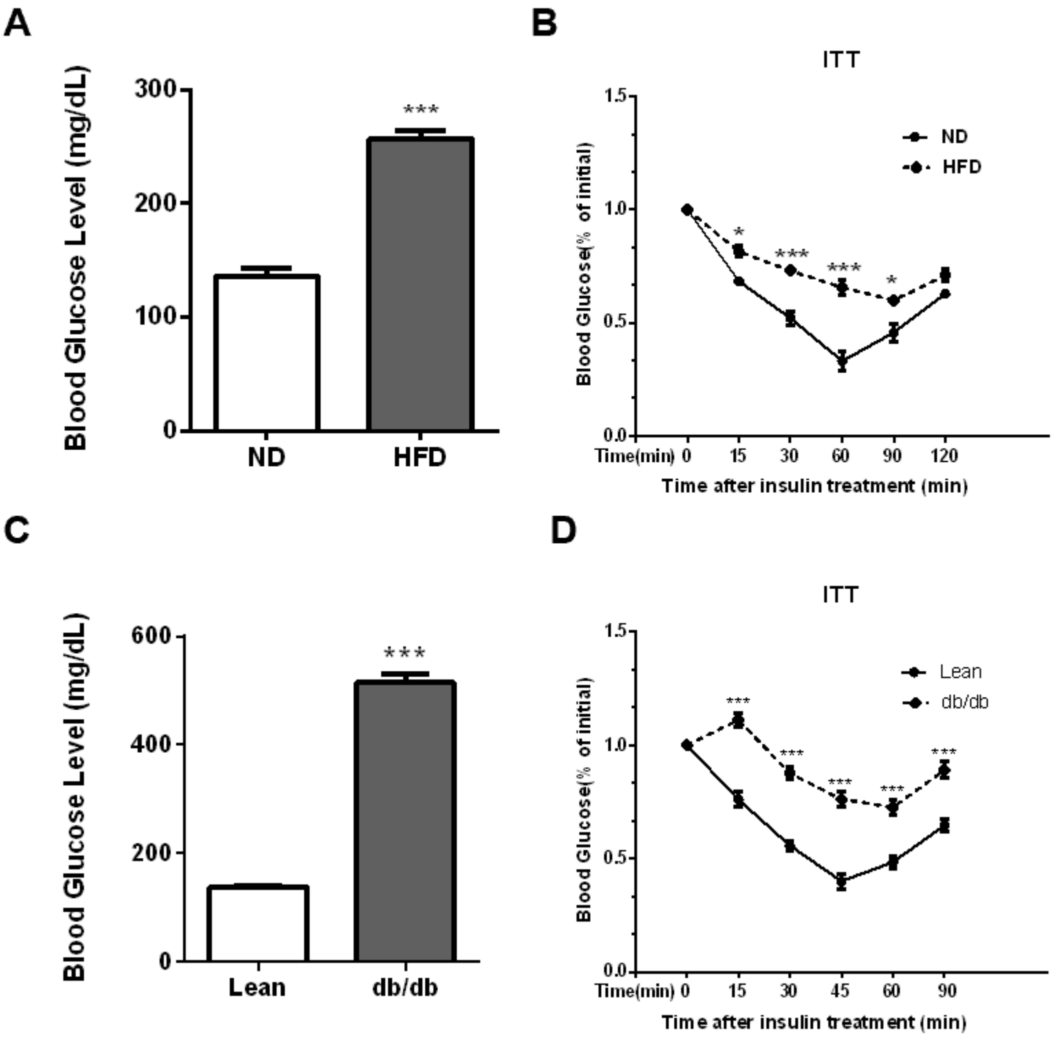

Figure S1. Diabetic parameters of 12-week HFD-fed mice and *db/db* mice. A-B: Examination of fasting blood glucose (A) and ITTs (B) in HFD-fed mice. C-D: Examination of fasting blood glucose (C) and ITTs (D) in *db/db* mice. *P < 0.05, ***P < 0.001.

Figure S2

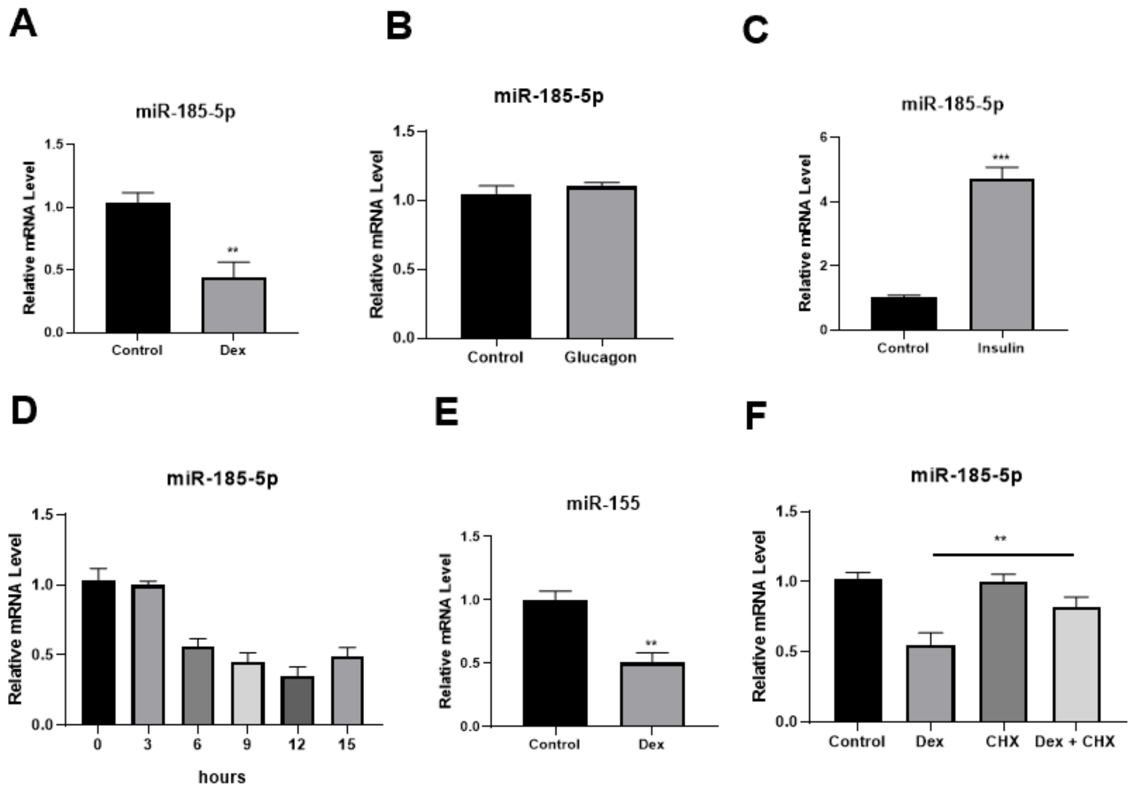

Figure S2. **Regulation of** miR-185-5p-5p **by glucocorticoid and insulin in hepatocytes**

A-C: Relative expression of miR-185-5p in Hep1-6 cells treated with dexamethasone (100nM, Dex, A), glucagon (10nM, B) or insulin (10nM, C) for 6 h. n=3 per group. **P < 0.01, ***P < 0.001.

D: Relative expression of miR-185-5p in MPHs treated with dexamethasone (100nM, Dex) for the indicated time course. E: Relative expression of miR-155 in MPHs treated with dexamethasone (100nM, Dex) for 2 h. F: Relative expression of miR-185-5p in MPHs treated with dexamethasone (100nM, Dex) with or without cycloheximide pretreatment. **P < 0.01.

Figure S3

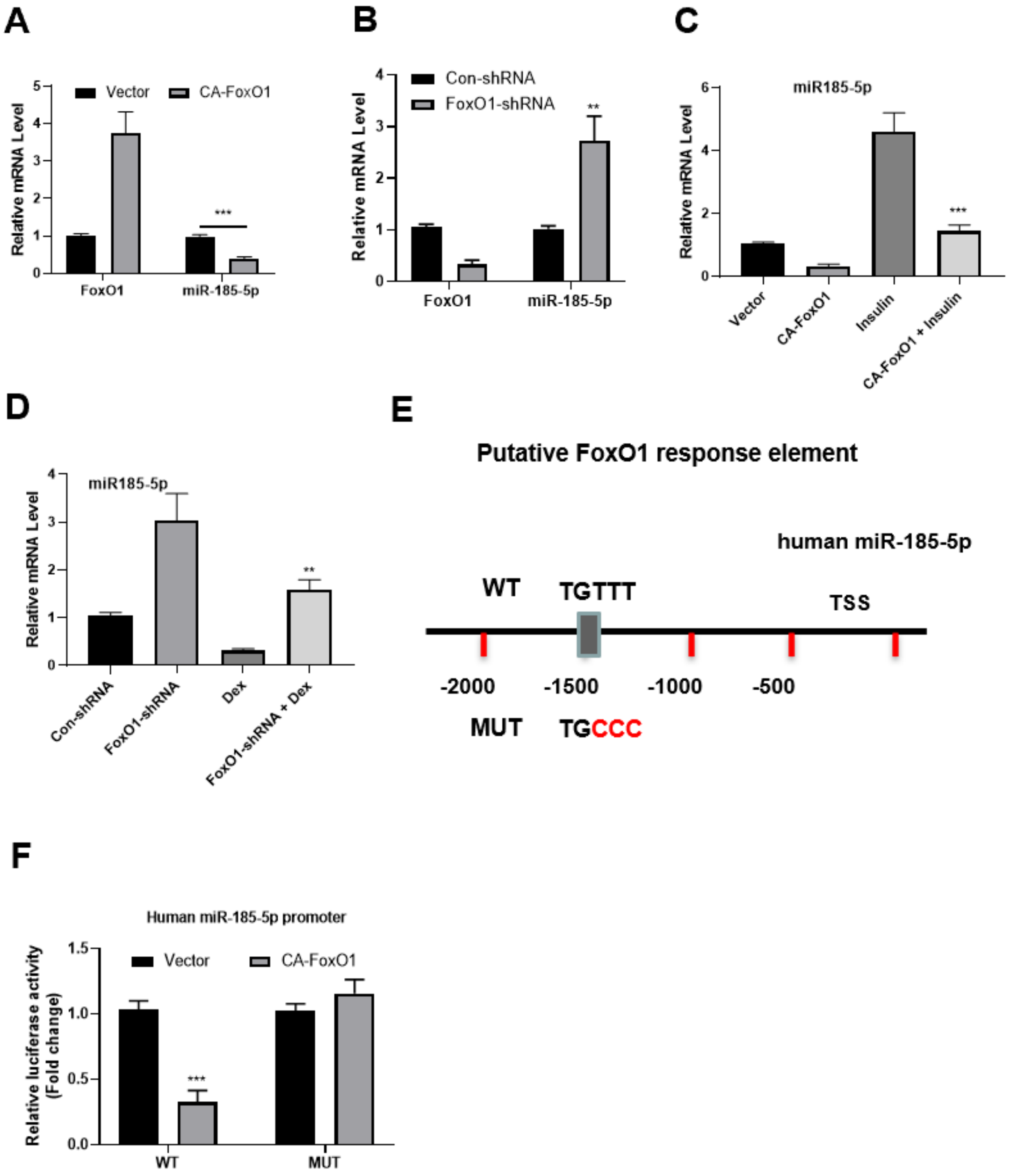

Figure S3. **Suppression of** miR-185-5p-5p **by FoxO1 in hepatocytes**

A: Hep1-6 cells were infected with adenovirus expressing a constitutively active FoxO1 (Flag-CA-FoxO1) or vector control. The mRNA expression levels of FoxO1 and miR-185 were measured by real-time PCR assay.

B: Hep1-6 cells were transfected with con-shRNA or shRNA against FoxO1. The mRNA expression levels

of FoxO1 and miR-185 were measured by real-time PCR assay.

C: Hep1-6 cells were infected with adenovirus expressing a constitutively active FoxO1 (Flag-CA-FoxO1) or vector control were treated with insulin (10nM, C) for 6 h. The mRNA expression levels of miR-185 were measured by real-time PCR assay.

D: Hep1-6 cells were transfected with con-shRNA or shRNA against FoxO1 were treated with dexamethasone (100nM, Dex) for 6 h. The mRNA expression levels of miR-185 were measured by real-time PCR assay.

E: Schematic diagram shows human miR-185 promoter and putative FoxO1 binding sites. TSS: transcription. The mutations were highlighted in red.

F: Relative luciferase activity of the firefly reporter containing the wt or mutant human miR-185 promoter was detected in HepG2 cells infected with adenovirus expressing a constitutively active FoxO1 (Flag-CA-FoxO1) or vector control.

**P < 0.01, ***P < 0.001.

Figure S4

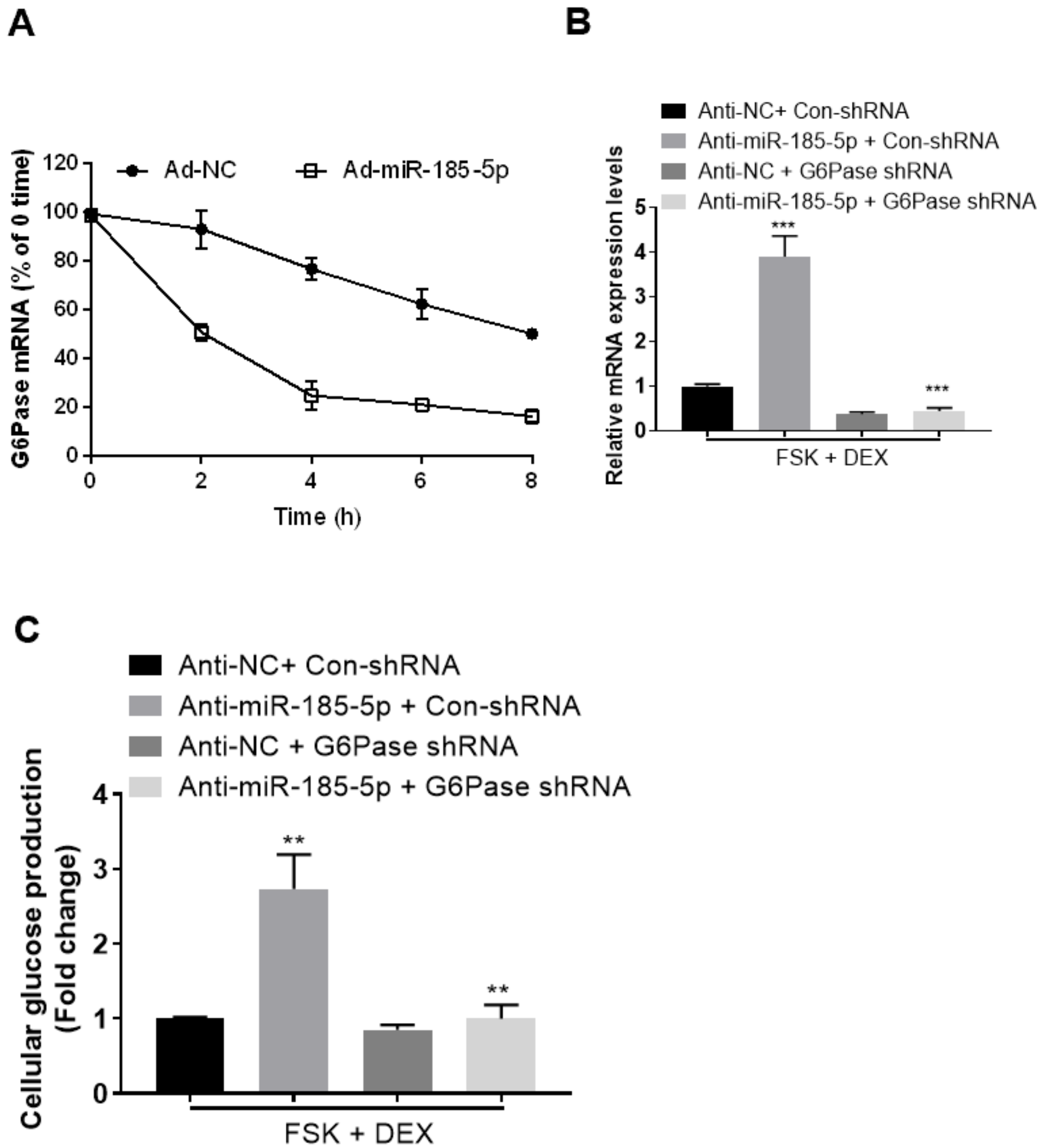

Figure S4. Mouse primary hepatocytes (MPHs) infected with Ad-NC or Ad-miR-185-5p were incubated with Actinomycin D (10 µg/ml). G6Pase/U6 mRNA ratio at 0 h was adjusted to 100% (A). MPHs were transfected with miR-185-5p antisense or negative control with or without G6Pase shRNA for 48 h and then treated with FSK (10 µM) and DEX (100 nm) for an additional 6 h. Then, the mRNA levels of G6Pase (B) and cellular glucose production were examined (C). $^{**}P < 0.01$, $^{***}P < 0.001$.

Figure S5

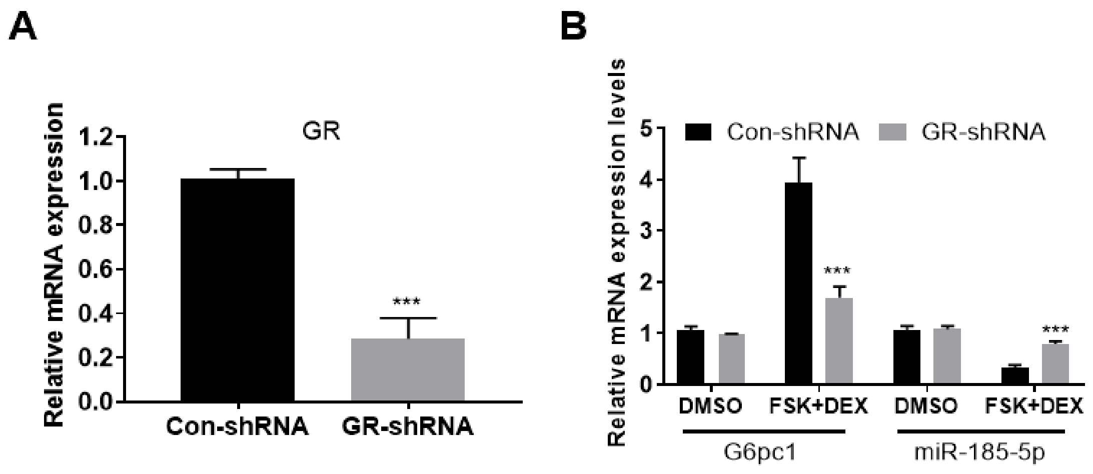

Figure S5. MPHs were transfected with GR-shRNA or control shRNA 48 h, the mRNA levels of GR were examined (A). MPHs were transfected with miR-185-5p antisense or negative control with or without GR-shRNA for 48 h and then treated with or without FSK (10 μM) and DEX (100 nm) for additional 6 hours. Then, the mRNA levels of G6Pase and miR-185-5p were examined (B). ***P < 0.001.

Figure S6

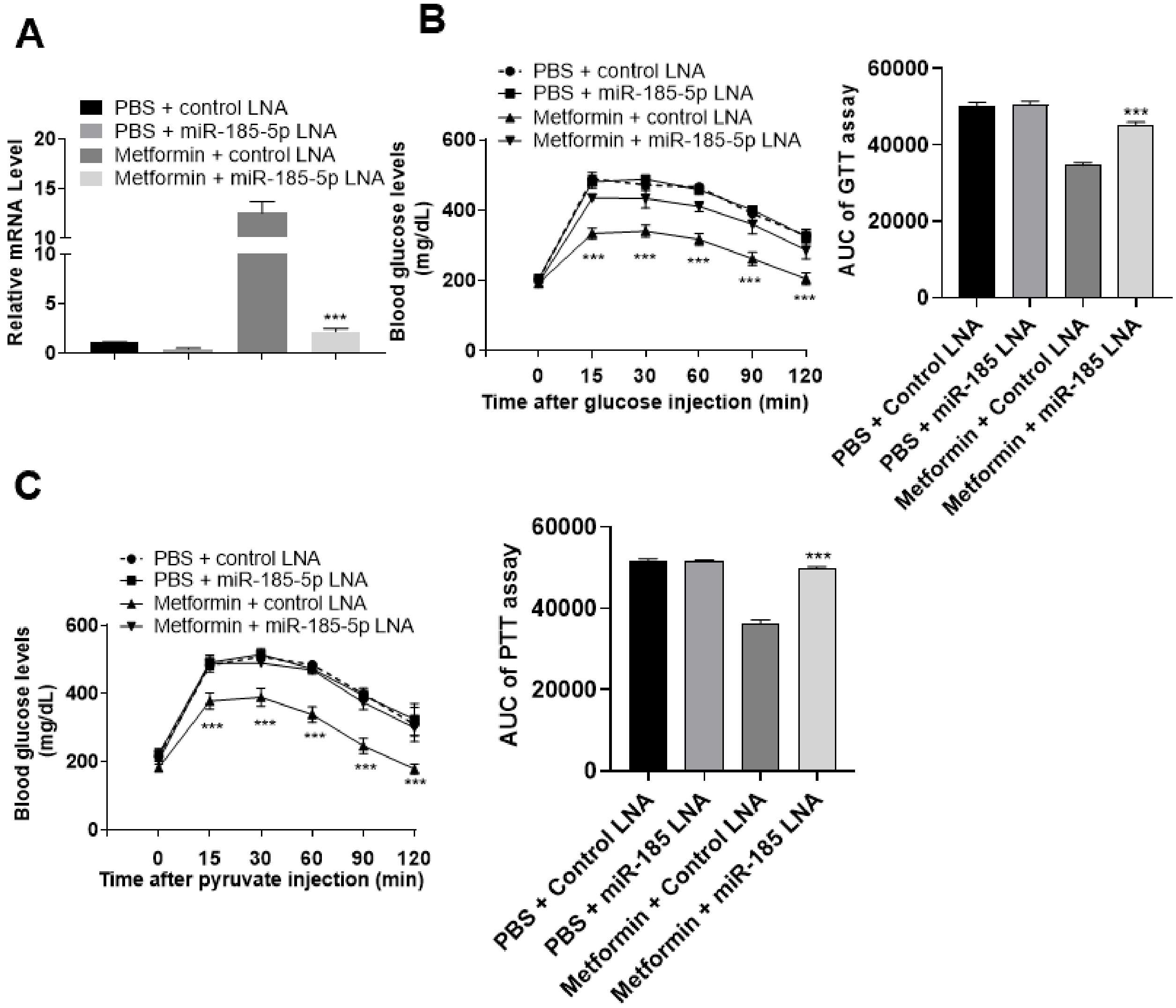

Figure S6. *db/db* mice were daily treated with metformin (200 mg/kg) or vehicle control by i.p. injection for 3 weeks, then injected with control LNA or miR-185-5p LNA for additional 6 days. The mRNA level of miR-185-5p in mice liver was detected (A). Glucose tolerance test (GTT) (B) and pyruvate tolerance test (PTT) (C) were determined, the AUC of glycemia was also calculate. ***P < 0.001.

Table S1

	Normal (n=20)	T2DM (n=20)	*P* value
Age, y	48.6 ± 6.4	49.1 ± 7.8	0.82
BMI, kg/m^2	22.9 ± 5.2	25.9 ± 4.4	0.056
WC, cm	83.5 ± 8.7	92.3 ± 7.4	0.0014
SBP, mmHg	114.3 ± 8.4	133.2 ± 12.5	<0.001
DBP, mmHg	78.5 ± 7.7	86.2 ± 6.1	0.0012
Lipids			
TC, mmol/L	4.87 ± 1.02	5.44 ± 1.34	0.138
Triglycerides, mmol/L	1.41 ± 0.85	1.93 ± 0.49	0.051
LDL-C, mmol/L	3.21 ± 0.98	3.09 ± 0.83	0.678
HDL-C, mmol/L	1.22 ± 0.34	1.27 ± 0.29	0.619
Fasting plasma glucose, mg/dL	86.77 ± 19.67	155.24 ± 30.53	< 0.001
HbA1c, %	5.12 ± 0.43	7.94 ± 0.62	< 0.001
Uric acid, mmol/L	243.5 ± 72.5	281.6 ± 82.7	0.129
CRP, mg/L	1.31 ± 0.47	1.75 ± 0.73	0.029
FFA, mmol/L	0.27 ± 0.14	0.48 ± 0.18	< 0.001
Cortisol, μg/dL	8.72 ± 1.69	12.94 ± 3.21	< 0.001

Table S1. General characteristics of type 2 diabetes and healthy patients. Examination of age, body mass index (BMI), waist circumference (WC), blood pressure, lipids, fasting plasma glucose, HbA1c, uric acid, c-reactive protein (CRP), free fatty acid (FFA) and cortisol.

Table S2

Ppargc1a-F	GTAAATCTGCGGGATGATGG
Ppargc1a-R	GGTGGAAGCAGGGTCAAAA
G6Pase-F	TGGTAGCCCTGTCTTTCTTTG
G6Pase-R	TTCCAGCATTCACACTTTCCT
PEPCK-F	ACACACACACATGCTCACAC
PEPCK-R	ATCACCGCATAGTCTCTGAA
GRα-F	ATCATGTTTGAGACCTTCAACA
GRα-R	CATCTCTTGCTCGAAGTCCA
miR-185-5p-F	TGCGGTGGAGAGAAAGGCAG
miR-185-5p-R	CTGCCTTTCTCTCCTCCGCA
U6-F	TGCGGGTGCTCGCTTCGGCAGC
U6-R	TGCGGGTGCTCGCTTCGGCAGC
Tk-F	ATGGAAGGTTACCATAAGCCAGA
Tk-R	CATGGCCCTTAGAGAGCACA
Lpk-F	GAACATTGCACGACTCAACTTC
Lpk-R	CAGTGCGTATCTCGGGACC
Khk-F	GTGGAGGCAACGCATCCAA
Khk-R	CAAGAGCAAGGGGTATCTCCC
Fasn-F	GGAGGTGGTGATAGCCGGTAT
Fasn-R	GGAGGTGGTGATAGCCGGTAT
Scd1-F	TTCTTGCGATACACTCTGGTGC
Scd1-R	CGGGATTGAATGTTCTTGTCGT
Acc1-F	GATGAACCATCTCCGTTGGC
Acc1-R	GACCCAATTATGAATCGGGAGTG

Table S2. List of primers used in this study.

Table S3

miRNA Information	Statistics & Regulation		
mature-miRNA	Fold Change	P-value	Regulation
mmu-miR-664-5p	2.8651	0.0341	up
mmu-miR-199b-3p	1.6571	0.0177	up
mmu-miR-200a-3p	1.7777	0.0345	up
mmu-miR-200b-3p	1.9459	0.0119	up
mmu-miR-221-3p	1.8387	0.0359	up
mmu-miR-222-3p	1.6666	0.0161	up
mmu-miR-23a-3p	1.724137931	0.0152	up
mmu-185-5p	0.1296	0.0075	down
mmu-129-3p	0.2528	0.0381	down
mmu-140-5p	0.2971	0.0092	down
mmu-191-5p	0.3632	0.0182	down
mmu-27a-5p	0.4486	0.0086	down
mmu-341-3p	0.5329	0.0202	down
mmu-99b-5p	0.6008	0.0048	down
mmu-let-7i-5p	0.6582	0.0117	down
mmu-miR-127-3p	0.509	0.0245	down
mmu-miR-129-5p	0.6666	0.0074	down
mmu-miR-129-5p	0.6666	0.0074	down
mmu-miR-140-3p	0.628	0.0334	down
mmu-miR-144-5p	0.5454	0.0074	down
mmu-miR-181d-5p	0.6666	0.0046	down
mmu-miR-1839-5p	0.6421	0.0223	down
mmu-miR-185-5p	0.6553	0.03454	down
mmu-miR-191-5p	0.6091	0.03943	down
mmu-miR-194-5p	0.6162	0.03551	down
mmu-miR-194-5p	0.6162	0.03551	down
mmu-miR-199a-3p	0.6034	0.0177	down
mmu-miR-199a-3p	0.6034	0.0176	down

Table S3. The miRNA expression in mouse liver using miRNA high throughput sequencing (up and downregulated).
